# Developing explicit customer preference models using fuzzy regression with nonlinear structure

**DOI:** 10.1007/s40747-023-00986-9

**Published:** 2023-02-21

**Authors:** Huimin Jiang, Xianhui Wu, Farzad Sabetzadeh, Kit Yan Chan

**Affiliations:** 1 School of Business, Macau University of Science and Technology, Macau, China; 2 Faculty of Business, City University of Macau, Macau, China; 3 School of Electrical Engineering, Computing and Mathematics Sciences, Curtin University, Bentley, WA Australia

**Keywords:** Explicit consumer preference models, Multi-objective optimization, Fuzzy regression with nonlinear structure, Sentiment analysis

## Abstract

In online sales platforms, product design attributes influence consumer preferences, and consumer preferences also have a significant impact on future product design optimization and iteration. Online review data are the most intuitive feedback from consumers on products. Using the value of online review information to explore consumer preferences is the key to optimize the products, improve consumer satisfaction and meet consumer requirements. Therefore, the study of consumer preferences based on online reviews is of great importance. However, in previous research on consumer preferences based on online reviews, few studies have modeled consumer preferences. The models often suffer from the nonlinear structure and the fuzzy coefficients, making it challenging to build explicit models. Therefore, this study adopts a fuzzy regression approach with a nonlinear structure to model consumer preferences based on online reviews to provide reference and insight for subsequent studies. First, smartwatches were selected as the research object, and the sentiment scores of product reviews under different topics were obtained by text mining on the product online data. Second, a polynomial structure between product attributes and consumer preferences was generated to investigate the association between them further. Afterward, based on the existing polynomial structure, the fuzzy coefficients of each item in the structure were determined by the fuzzy regression approach. Finally, the mean relative error and mean systematic confidence of the fuzzy regression with nonlinear structure method were numerically calculated and compared with fuzzy least squares regression, fuzzy regression, adaptive neuro fuzzy inference system (ANFIS) and K-means-based ANFIS, and it was found that the proposed method was relatively more effective in modeling consumer preferences.

## Introduction

Since the information age, people's lifestyles have changed dramatically and they are using the internet to socialize, shop and entertain themselves. The number of social media users and online shoppers worldwide has continued to grow in recent years due to the impact of COVID-19 [1]. Not only are people accessing more data and information via the internet, they are also sharing their opinions and comments on the platforms as they do so. These online reviews often contain a great deal of information about the needs and values of consumers, which not only reflect their wishes, but also reveal their innermost desires. Thus, uncovering their preference is essentially important.

Previous studies have attempted to model consumer preferences to find the link between consumer preferences and product attributes based on these online reviews. However, some research issues have been found. First, the highly nonlinear nature of the relationships needs to be addressed in the customer preference models [2]. Second, the fuzziness existing in online reviews need to be considered in the models. Third, the models developed in the previous studies have the low explanatory nature and are unable to display an explicit model. Therefore, to solve the above issues, a fuzzy regression with nonlinear structure approach is proposed in this paper to build an explicit consumer preference model based on online reviews. The method requires sentiment analysis of online reviews to derive sentiment scores for the extracted consumer preferences, in the hope of addressing the pain points of nonlinearity and fuzziness in relationships, and no explicit model that exists in current research. The main contributions of this paper include: first, the paper proposed a novel approach for modeling customer preferences based on online reviews, which combine the multi-objective chaos optimization (MCO) and the fuzzy regression method to solve the problems of fuzziness and high nonlinearity that arise when modeling. Second, in this approach, a new MCO algorithm that uses the mean relative error (MRE) and mean systematic confidence (MSC) as the objective functions is proposed to build the polynomial structures of the model, which is not only more capable of global retrieval than traditional algorithms, but also has the characteristics of "ergodicity, randomness and regularity", which can simultaneously prevent the chaotic motion from falling into local minima in practical applications [3]. The way of generating the polynomial structure is a new idea proposed to capture the nonlinearity of the modeling and display the nonlinear structure explicitly. Thirdly, the study applied fuzzy regression [4], a method that models fuzzy relationships by applying fuzzy functions to derive the relationships with fuzzy parameters. This method is better suited to the fuzzy relationship between consumer preferences and product attributes, and the resulting correlation parameters are more accurate.

This paper is organized as follows. "Related works" provides the content of the literature review, which reviews the relevant research on sentiment analysis and consumer preference modeling by previous scholars. "Research methodology" describes the proposed fuzzy regression with nonlinear structure method and gives an explanation of how the method can be applied to modeling consumer preference. "Implementation" is an experimental section, which is an example of modeling the product design attributes and the mined consumer preferences based on online reviews of smartwatch products. "Validation" gives a validation experiment in which the experimental results are compared with those of the other four methods. "Discussion" provides a discussion of this study. Finally, in "Conclusion", the experiments are summarized and presented in a prospective manner.

## Related works

Based on the introduction, the related research works are provided as follows. Sentiment analysis, often referred to as opinion mining, aims to mine sentiment from textual data information, which not only detects the public's main opinions on products, things, and hot events but also provides valuable information that can help in decision-making [5]. As an important research direction in natural language processing, text sentiment analysis has been successfully applied to the field of online reviews. For many online platforms, online review information not only influences consumers' purchasing behavior, but also reveals consumer preferences and how much they like the different features of a product [6]. Therefore, it is important to collect and analyze the review data to quickly identify problems with the product and to help in the subsequent evaluation and optimization of the product design. A supervised machine learning approach for sentence-level adaptive text extraction and mining has been proposed [7] to extract consumer needs by analyzing user-generated online product reviews. Researchers Chen et al. [8] have developed an ontology learning system for customer needs representation in product development. Kang and Zhou proposed a method called "RubE" in the literature to extract product features [9]. This unsupervised rule-based extraction method can also tap into the subjective and objective features of consumers from online reviews, providing a new way of thinking about the role of different product features in personalized marketing recommendations. To find the reasons for consumers' positive or negative emotions when confronted with different product features, researchers proposed OPINSTREAM, a framework for extracting product features from online reviews, which can further monitor the implicit product features [10]. Jiao et al. broke with tradition using a framework that combines affective lexicons mixed with a rough-set technique to study online reviews and build a feature model that can predict the sentence sentiments of product features [11]. In addition, some scholars have automated the design of products that predict consumer preferences through a text mining and Kansei Engineering (KE) approach [12], which not only reduces the design task for product designers but also provides intelligent operations for mining consumer preferences. In addition to the above-related studies, there are also some studies that mine product feature information based on the analysis of social media data. Pitchayaviwat [13] conducted the feature extraction based on product information extracted from social media in his study in 2016, and the performance of two clustering algorithms, K-means and self-organization map (SOM), was evaluated experimentally. Tucker and Tuarob proposed a knowledge discovery in databases (KDD) model based on social media numbers to help predict product market adoption and longevity by mining product information in social reviews [14]. In addition, they developed a method to automatically identify users' product characteristics based on social media data [15] and designed an automatic quantification of functional interactions for modules that can extract textual information to mine key users and their consumer preferences for products [16]. Ordinal classification approach [17], which is applied to the identification of product features and the completion of product feature weighting, can provide useful references in product feature design while learning features. Other studies based on online reviews have employed a number of methods to identify consumer preferences. For example, Rai [18] has independently classified the product attributes based on online reviews and identified the importance of different attributes in product design. In addition, the Bayesian sampling method, a commonly used method, can also successfully extract product feature information from a large amount of data information [19]. And Yang et al. [20] considered the different conditions of local and global information of data and combined the local and global information for product feature extraction and feature ranking in viewpoint mining of textual information. Zhou and Liao [21] proposed a dynamic evaluation framework for hotel customer preferences through sentiment analysis on online reviews.

Previous studies have proposed some approaches to model the relationships between customer preferences and product design attributes. Wang et al. [22] have used the User-Generated Content (UGC) based on online reviews and collected product attributes from the UGC to construct consumer preference models. However, such models do not have a specific explicit structure. A multi-objective particle swarm optimization (PSO) approach [23] has emerged, in which if so rules are built to explore the relationship between product design attributes and consumer preferences [24]. Wang and Zhou [25] applied the fuzzy weighted association rule mining method to mine the association rules between user preferences and product features. However, these approaches are limited by the if so rules and fail to dig deeper into the association between consumer preferences and product design attributes. As a result, scholars have gradually realized that the current research methods cannot meet the practical development needs, and there are still many shortcomings in numerous methods such as statistical linear regression [26], partial least squares analysis [27], belief rule-based square theory [28], and artificial neural networks [29]. For example, the modeling process often suffers from fuzziness that is difficult to resolve due to the small number of data sets and the involvement of subjectivity in the data information. As a result, many scholars have also started to study the problems concerning fuzziness, which has led to fuzzy theory-based methods such as fuzzy rule-based methods [30], fuzzy inference methods [31], a nonlinear possibilistic regression method [32] and fuzzy linear regression [33]. A flexible fuzzy regression-data envelopment analysis algorithm was introduced for modeling customer preferences with new product design [34]. A dynamic evolving neural-fuzzy inference system was applied for the modeling of variational customer preferences for the design of hair dryers [35]. Yakubu et al. [36] proposed a multigene genetic programming-based fuzzy regression approach to develop customer preference models based on online reviews. But in addition to the need to address the problem of vagueness, scholars have found that when modeling product design attributes and consumer preferences, the relationships are often highly nonlinear. As a result, the polynomial structure based on fuzzy regression methods has emerged, for example, fuzzy regression based on forward selection [37], stepwise fuzzy regression [38], fuzzy regression based on genetic programming [39], and chaos-based fuzzy regression [40].

To summarize, three key conditions need to be met to explore the link between consumer preferences and product design attributes in depth: the nonlinear model with polynomial structure needs to be constructed; the fuzziness in the model building needs to be explored further to ensure that the fuzzy coefficient for each item in the polynomial structure is identified; the developed models should be explicit and explainable. In view of these, a novel approach based on online reviews needs to be developed to meet the requirements for modeling the relationships between product design attributes and consumer preferences.

## Research methodology

**Fig. 1:**
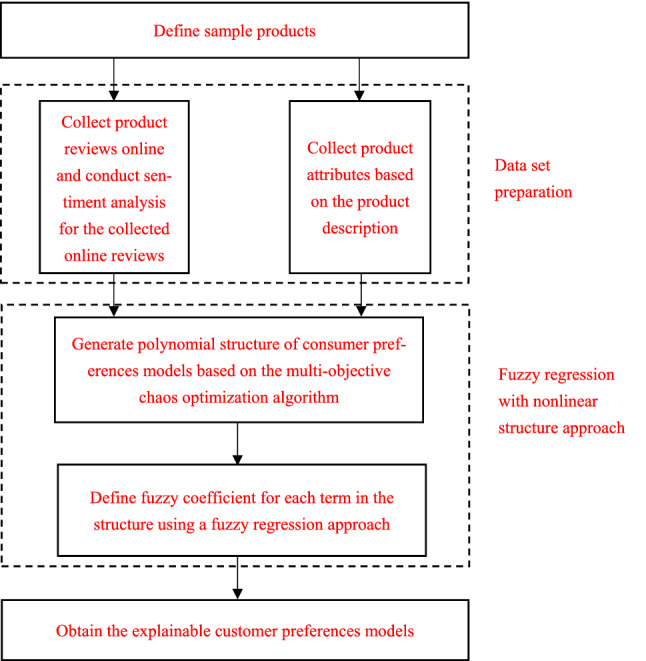
A flowchart of the proposed approach

To solve the above research issues, this paper adopts a fuzzy regression with nonlinear structure approach to build a consumer preference model based on online reviews. The flowchart of the proposed approach is shown in Fig. 1. The first step in building the model is the preparation of the dataset based on sentiment analysis, followed by the determination of the polynomial structure of the consumer preference model based on MCO. Then, the fuzzy regression method is applied to determine and assign the fuzzy coefficients of the model. Finally, the explicit consumer preference model can be determined based on the generated polynomial structure and fuzzy coefficients. The main steps and principles of the algorithm are as follows.

### Data set preparation based on sentiment analysis

In this paper, a sample of online reviews of 10 mainstream smartwatches was collected from the Amazon platform and stored in Excel. First, the raw data were pre-processed, mainly for data cleaning, text segmentation, and deactivation filtering. Second, the RINGDATA platform was used for topic classification and sentiment score calculation, mainly using the latent Dirichlet allocation (LDA) topic model and the weight-based sentiment score calculation. Through the topic categorization, five main categories were identified, with the category names "Quality", "Customer experience", "Function ", "Smart" and "Affordable". The sentiment scores of each category were calculated using online reviews, representing the sentiment scores of consumer preferences and the modeling output. Data on the design attributes of the smartwatches were also collected, which are used as the inputs to the experiment.

### Polynomial structure of consumer preference models

After the data preparation, a polynomial structure needs to be set up to satisfy the modeling of product design attributes and consumer preferences. The inputs to the model are the product design attributes and the output is the mined consumer preference. In this model (1), ${x}_{{i}_{j}}$ is the ${i}_{j}th$ independent variable, $i_{j} = 1, \ldots ,N$ and $j=1,\cdots d$. *N* and *d* are the number of inputs**.** $\widetilde{A}$ denotes the fuzzy coefficients generated using the fuzzy regression method, where the fuzzy coefficient of each structure is given by the combination of the central value of fuzzy coefficient ${a}^{c}$ and the corresponding expansion of the fuzzy coefficients ${a}^{s}$. $\widetilde{y}$ is the dependent variable. An example of the model is shown in (1).

1 $$ \tilde{y} = f_{{{\text{NR}}}} \left( x \right) = \tilde{A}_{0} + \sum\limits_{{i_{1} = 1}}^{N} {\tilde{A}_{{i_{1} }} x_{{i_{1} }} } + \sum\limits_{{i_{1 = 1} }}^{N} {\sum\limits_{{i_{2} = 1}}^{N} {\tilde{A}_{{i_{1} i_{2} }} x_{{i_{1} }} x_{{i_{2} }} } } + \sum\limits_{{i_{1} = 1}}^{N} {\sum\limits_{{i_{2} = 1}}^{N} {\sum\limits_{{i_{3} = 1}}^{N} {\tilde{A}_{{i_{1} i_{2} i_{3} }} x_{{i_{1} }} x_{{i_{2} }} x_{{i_{3} }} } } } + \cdots \sum\limits_{{i_{1} = 1}}^{N} \cdots \sum\limits_{{i_{d = 1} }}^{N} {\tilde{A}_{{i_{1} \cdots i_{d} }} } \prod\limits_{j = 1}^{d} {x_{j} } , $$

$$ \tilde{A}_{0} = \left( {a_{0}^{c} ,a_{0}^{s} } \right),\tilde{A}_{1} = \left( {a_{1}^{c} ,a_{1}^{s} } \right),\tilde{A}_{2} = \left( {a_{2}^{c} ,a_{2}^{s} } \right), \ldots ,\tilde{A}_{N} = \left( {a_{N}^{c} ,a_{N}^{s} } \right), $$

$$ \tilde{A}_{11} = \left( {a_{11}^{c} ,a_{11}^{s} } \right),\tilde{A}_{12} = \left( {a_{12}^{c} ,a_{12}^{s} } \right),\tilde{A}_{13} = \left( {a_{13}^{c} ,a_{13}^{s} } \right), \ldots ,\tilde{A}_{NN} = \left( {a_{NN}^{c} ,a_{NN}^{s} } \right), $$

$$ \tilde{A}_{111} = \left( {a_{111}^{c} ,a_{111}^{s} } \right),\tilde{A}_{112} = \left( {a_{112}^{c} ,a_{112}^{s} } \right),\tilde{A}_{113} = \left( {a_{113}^{c} ,a_{113}^{s} } \right), \ldots ,\tilde{A}_{NNN} = \left( {a_{NNN}^{c} ,a_{NNN}^{s} } \right), \ldots ,\tilde{A}_{N \cdots N} = \left( {a_{N \cdots N}^{c} ,a_{N \cdots N}^{s} } \right). $$

### Determination of the polynomial structure based on the MCO

The polynomial structure of the customer preferences models is determined based on the MCO algorithm. The concept of Chaos Optimization Algorithm (COA) first appeared in 1997 [41] and the method is mainly useful for solving the combinatorial optimization problems, which can be used to solve nonlinear optimization problems. It uses the "randomness", "ergodicity" and "regularity" of chaotic variables to search the solution space (the range of solutions transformed by the variables in the chaotic space). The process can find the optimal global solution after several iterations [42]. The whole search process can be divided into four main steps.

**Step 1.** First, the chaotic variables in the COA algorithm is applied here as the Logistic mapping (2), where ${c}_{k}$ denotes the *k*th iteration value of the chaotic variable *c*, which will output values in the range [0,1] and is characterized by randomness and traversal within the interval. $\mu$ is the control covariates, and $\mu$ ∈ (0,4), when $\mu$ = 4, the best chaotic sequence occurs.

2 $$ c_{k + 1} = f\left( {c_{k} } \right) = \mu c_{k} \left( {1 - c_{k} } \right) . $$

**Step 2.** According to (3), by mapping the chaotic variable ${c}_{k}$, the optimization variable ${q}_{k}$ is obtained. In this process, *a* is the minimum value of ${q}_{k}$ and *b* is the maximum value of${q}_{k}$. Therefore, the traversal range of the optimization variables is [*a*, *b*].

3 $$ q_{k} = a + \left( {b - a} \right)c_{k} . $$

**Step 3.** Iterate repeatedly to obtain the value of the new solution.

**Step 4.** Keep searching for the optimal solution within the local area before outputting the optimal value until the termination condition is satisfied.

Based on MCO, a polynomial structure can be obtained for the consumer preference model, and the structure ${q}_{n}$ is given by the input ${x}_{1}$*,* ${x}_{2},\dots {,x}_{N}$ and the operator symbols ("+"), ("*") between the inputs. In addition, the vector ${q}_{k}$ is shown in (4), where $\mathrm{Ne}$ is the number of elements within ${q}_{k}$ and $\mathrm{Ne}$ is usually an odd number, which is set to 13 in this study.

4 $$ q_{k} = \left[ {q_{k}^{1} ,q_{k}^{2} , \ldots ,q_{k}^{{{\text{Ne}}}} } \right]. $$

The structure of the chaos variable is further described here. The odd elements of the structure can be represented as $\left[ {q_{k}^{1} ,q_{k}^{3} , \ldots ,q_{k}^{{{\text{Ne}}}} } \right]$. Each element is an integer and the value is in the range [1,4], which means the *i*th input ${x}_{i}$ in the model. The elements of the structure that are even can be represented as $\left[ {q_{k}^{2} ,q_{k}^{4} , \ldots ,q_{k}^{{{\text{Ne}} - 1}} } \right]$ which is used to obtain the arithmetic operation symbols. A value of 0 represents the operation of addition, and a value of 1 represents the operation of multiplication. Thus, if we take ${q}_{n}=\left[\mathrm{1,0},\mathrm{2,1},\mathrm{3,1},\mathrm{2,0},\mathrm{4,0},\mathrm{3,1},1\right]$ as an example, the polynomial structure can be expressed as ${x}_{1}$+${x}_{2}^{2}{x}_{3}$+${x}_{4}$+${x}_{1}{x}_{3}$.

In the MCO process, the study also applied two metrics that can measure the reliability of fuzzy regression models [43], namely MRE and MSC. In the expression for MRE (5)*,* the $ND$ denotes the number of data sets; the fuzzy number ${\tilde{y}}_{l}$ = (${\tilde{y}}_{i}{ }^{R}$, ${\tilde{y}}_{l}^{c}$, ${\tilde{y}}_{i}{ }^{L}$), represents the ith predicted output, where ${\tilde{y}}_{i}{ }^{R}$ is the right spread, ${\widetilde{y}}_{l}^{c}$ is the center value, and ${\tilde{y}}_{i}{ }^{L}$ is the left spread. In the MSC expression (6), the smaller the denominator $\left|{\tilde{y}}_{i}{ }^{R}-{\tilde{y}}_{i}{ }^{L}\right|$ means the smaller fuzzy spread; the numerator ${\mu }_{{\tilde{y}}_{l}}\left({y}_{i}\right)$ represents the membership degree of ${y}_{i}$ to ${\widetilde{y}}_{l}$, which is calculated from (7). ${\widetilde{{y}_{i}}}^{s}$ is the spread of$\widetilde{{y}_{i}}$. In summary, the smaller values of MRE denote the more reliable developed models because they produce smaller errors. In contrast, the larger values of MSC mean more reliable models as they will produce a stronger degree of systematic feasibility.

5 $$ {\text{MRE}} = \frac{1}{{{\text{ND}}}}\sum\limits_{i = 1}^{{{\text{ND}}}} {\frac{{\left| {\tilde{y}_{l}^{c} - y_{i} } \right|}}{{y_{i} }}} , $$

6 $$ {\text{MSC}} = \sum\limits_{i = 1}^{{{\text{ND}}}} {\frac{{\mu_{{\tilde{y}}} \left( {y_{i} } \right)}}{{\Delta \left( {\tilde{y}_{i} } \right)}}} = \sum\limits_{i = 1}^{{{\text{ND}}}} {\frac{{\mu_{{\tilde{y}_{l} }} \left( {y_{i} } \right)}}{{\left| {\tilde{y}_{i} ^{R} - \tilde{y}_{i} ^{L} } \right|}}} , $$

7 $$ \mu_{{\tilde{y}_{l} }} \left( {y_{i} } \right) = \left\{ {\begin{array}{*{20}r} \hfill 1 & \hfill {y_{i} = \tilde{y}_{l}^{c} } \\ \hfill {1 - \frac{{\left| {y_{i} - \tilde{y}_{l}^{c} } \right|}}{{\tilde{y}_{l}^{s} }}} & \hfill {\tilde{y}_{l}^{c} - \tilde{y}_{l}^{s} < y_{i} < \tilde{y}_{l}^{c} + \tilde{y}_{l}^{s} } \\ \hfill 0 & \hfill {\text{ otherwise }} \\ \end{array} } \right.. $$

The process of generating the final model involves constant iterative updating of the polynomial structure, that is, the process of Step 2 and Step 3. During the process, the Pareto optimal solution needs to be obtained by comparing the MRE and MSC, which are denoted by ${\mathrm{OF}}_{1}$ and ${\mathrm{OF}}_{2}$, respectively. A fitness set ${F}_{S}$ is expressed as${F}_{S}=\left\{{\mathrm{OF}}_{1},{\mathrm{OF}}_{2}\right\}$. For a minimization optimization problem, if it satisfies ${\mathrm{OF}}_{i}(A)\le {\mathrm{OF}}_{i}(B)$, for all $i\in \{\mathrm{1,2}\}$ and ${\mathrm{OF}}_{j}(A)<{\mathrm{OF}}_{j}(B)$, for some $j\in \{\mathrm{1,2}\}$, then it means that solution B is dominated by solution A. In other words, when encountering a maximization optimization problem, if ${\mathrm{OF}}_{i}(A)\ge {\mathrm{OF}}_{i}(B)$, for all $i\in \{\mathrm{1,2}\}$ and ${\mathrm{OF}}_{j}(A)>{\mathrm{OF}}_{j}(B)$, for some $j\in \{\mathrm{1,2}\}$ are satisfied, the solution *B* is dominated by the solution *A*. Thus, the Pareto optimal solution can be obtained by finding a solution that is not dominated by other solutions.

### Determining the central value and expansion of fuzzy coefficients

Once the nonlinear structures are generated, it is time to start identifying the fuzzy coefficients of each term in the structure, where the fuzzy regression method [44] was applied. In the optimization model, the objective function is set to minimize the total fuzziness (8), and the constraints are described as shown in (9) and (10).

8 $$ {\text{Min}}J = \sum_{j = 0}^{NC} \left( {a_{j}^{S} \sum\limits_{i = 1}^{{{\text{ND}}}} {\left| {x_{j}^{^{\prime}} \left( i \right)} \right|} } \right), $$

9 $$ \sum\limits_{j = 0}^{{{\text{NC}}}} {a_{j}^{c} x_{j}^{^{\prime}} \left( i \right)} + \left( {1 - h} \right)\sum\limits_{j = 0}^{{{\text{NC}}}} {\left( {a_{j}^{s} \left| {x_{j}^{^{\prime}} \left( i \right)} \right|} \right)} \ge y_{i} ,i = 1,2, \ldots ,{\text{ND}}, $$

10 $$ \sum\limits_{j = 0}^{{{\text{NC}}}} {a_{j}^{c} x_{j}^{^{\prime}} \left( i \right)} - \left( {1 - h} \right)\sum\limits_{j = 0}^{{{\text{NC}}}} {\left( {a_{j}^{s} \left| {x_{j}^{^{\prime}} \left( i \right)} \right|} \right)} \le y_{i} ,i = 1,2, \ldots ,{\text{ND,}} $$

$$a_{j}^{s} \ge 0,a_{j}^{c} \in R,j = 0,1,2, \ldots ,{\text{NC}}$$

$$x_{0}^{^{\prime}} \left( i \right) = 1\, {\text{for all}} \, i \, \text{and}\, 0 \le h \le 1 .$$

In (8), the $J$ represents the total fuzziness; and $ND$ is the number of data sets. $NC+1$ is the number of terms of the polynomial structure; and ${x}_{j}^{\mathrm{^{\prime}}}(i)$ is the jth transformed terms in the models of the *i*th data set. In constraints (9) and (10), $h$ represents the extent to which the fuzzy model fits the actual data. This set of constraints ensures that ${\mu }_{{\tilde{y}}_{l}}\left({y}_{i}\right)\ge h$, *i* = 1,2, ⋯ ND which means each output ${y}_{i}$ has at least $h$ degree satisfying the condition.

## Implementation

In the real industry, the proposed approach can be used to analyze consumer goods' customer preferences, which have online reviews. Based on the description of the proposed approach in "Research methodology", a real case study on the products of the smartwatch is used to illustrate and evaluate the proposed approach. Online review data of 10 smartwatch products with a time of 2 years were collected as samples from Amazon platform using web crawler technology. The sample data were first cleaned and sentiment scores were calculated, where the 10 sample products were represented by 1–10, and the online reviews were analyzed for sentiment using the RINGDATA platform.

To explore the valuable information in the online reviews, in the preliminary sentiment analysis research process, we used word frequency statistics, LDA topic classification, and sentiment score calculation method to divide the review data into 5 categories and calculate the sentiment score results accordingly. These 5 sets of data represent the 5 categories of "Quality", "Customer experience", "Function", "Smart" and "Affordable". For example, in the "Customer experience" category, the words such as easy, useful, fitness, powerful, and comfortable often appear in the review messages. Therefore, the sentiment score for each review is calculated based on the sentiment word, word frequency, and topic relevance, as shown in the table below (Table 1).

**Table 1:** Product design attributes and sentiment scores for smartwatches

Products	Attributes of smartwatch				Customer experience
	Screen size (inch)	Volume (cm)	Weight (g)	Service Time (day)	Sentiment Scores
1	1.27	1.9440	49.90	14	0.273995163
2	1.28	23.2320	99.05	1	0.03210269
3	1.30	25.9604	36.30	8	0.053173582
4	1.10	20.2185	36.85	7	0.153304441
5	1.40	0.1202	31.00	7	0.028606627
6	1.43	0.9847	30.80	9	0.215711148
7	1.20	1.3294	43.10	7	0.258940243
8	1.33	19.8887	99.00	7	0.265835168
9	1.30	0.9075	43.90	10	0.183561497
10	1.30	117.6154	50.00	10	0.031180613

In the process of collecting online review data of smartwatch products, this paper also collected the relevant product attributes that may affect the final preference of consumers, and found that there are four product attributes that may affect the sentiment score of "Customer experience", namely Screen Size, Volume, Weight and Service Time. They represent the display size, product size, weight, and battery life of a smartwatch with the unit of inch, cm, gram and day, respectively.

After collecting and organizing the basic information, we tried to build the model for this experiment using fuzzy regression with nonlinear structure method. In this paper, four product attributes were used as inputs and the sentiment score of customer experience was used as an output to build a fuzzy model with the polynomial structure. The model was built using Matlab programming software, where the number of iterations of the model was set to 100; the number of elements in the chaotic variables was set to 13; the range of odd elements in the optimization variables was [1,4], and the range of even elements was [0,1]; for the problem of determining the h-value in fuzzy regression, experiments were conducted in the range of [0,1] for different h-values. The h-value related to the minimum modeling error is 0.1. After setting up the model, it can be run using Matlab, and the relevant results can be obtained by continuous iteration. In this paper, validation 1 is used as an example to demonstrate the results. The optimal solution $q$ = [2,0,4,0,3,1,2,1,3,0,4,1,4] can be obtained through iteration, and based on the results of this data, the model polynomial structure can be initially constructed as ${x}_{2}$**+**${x}_{4}$**+**${x}_{3}^{2}{x}_{2}$**+**${x}_{4}^{2}$**.** After that, fuzzy regression is used to determine the fuzzy coefficients for each item in the structure. The model for “Customer experience”, in the final validation 1 experiment, takes the form of *y* = (− 0.1477, 0.2297) + (− 0.0036, 4.3 × ${10}^{-4}$) ${x}_{2}$+(0.0617, 0) ${x}_{4}$ + (8.9 × 10^–7^, 6.1 × ${10}^{-7}$) ${x}_{2}{x}_{3}^{2}$+(− 0.0028, 0) ${x}_{4}^{2}$. The coefficients of all terms in the model are fuzzy, and the polynomial structure contains first-order terms ${x}_{2}$ and ${x}_{4}$ as well as the interactive terms ${x}_{3}^{2}{x}_{2}$ and ${x}_{4}^{2}$.

The modeling process of customer preference for the smartwatch products was implemented and the relationships between customer experience and screen size, volume, weight, and service time were established. The model not only confirms the nonlinearity and fuzziness between product attributes and consumer preferences but also provides a basis for the future prediction of consumer preferences in terms of preference sentiment scores. Based on the above-generated model, if the new smartwatch is designed, the corresponding sentiment score of customer experience with the new settings of product design attributes can be calculated for the reference of the product company. In addition, the best settings of product design attributes can be obtained based on the optimization of the generated model with the maximization of the value of the customer preference.

## Validation

To further verify the effectiveness of the proposed method, five validation tests were taken, and the proposed method was experimentally compared with fuzzy least squares regression (FLSR), fuzzy regression (FR), adaptive neuro fuzzy inference system (ANFIS) and K-means-based ANFIS based on the MRE and MSC values. In K-means-based ANFIS, the method of K-means is introduced into ANFIS to determine the membership function of inputs for ANFIS. Firstly, the dataset was divided. The experiment divided the dataset of ten products collected into validation and training sets. If two of the ten product datasets are used as validation sets, then the other eight datasets are used as training sets. Among products 1, 2, 3, 4, 5, 6, 7, 8, 9 and 10, products 1 and 2, 3 and 4, 5 and 6, 7 and 8, 9 and 10 will be used in turn as validation test datasets for non-reuse experiments. The next step is the basic setup of the experiments, which are all parameterized as described in Section IV. Then, the consumer preference models can be generated. Since ANFIS and K-means-based ANFIS have black box problems, the corresponding models cannot be shown explicitly. The comparison of the generated models presented for the three methods is shown in Table 2.

**Table 2:** The developed models based on three approaches in the five validations

*Validation1*	
FLSR	*y* = (0.0839, 0.0598) + (0.0597, 0.0594) ${x}_{1}$ + (− 0.0002, 0) ${x}_{2}$+ (0.0002, 0) ${x}_{3}$+ (0.0007, 0.0033)${x}_{4}$
FR	*y* = (0.3309, 0.8135) + (− 0.3393, 0.1940) ${x}_{1}$+ (− 0.0025, 0) ${x}_{2}$+ (0.0037, 0) ${x}_{3}$+ (0.0195, 0.0002)${x}_{4}$
FR with nonlinear structure	*y* = (− 0.1477, 0.2297) + (− 0.0036, 4.3 × ${10}^{-4}$) ${x}_{2}$+ (0.0617, 0) ${x}_{4}$+ (8.9 × 10^–7^, 6.1 × ${10}^{-7}$) ${x}_{2}{x}_{3}^{2}$+ (− 0.0028, 0)${x}_{4}^{2}$
*Validation2*	
FLSR	*y* = (0.0907, 0.0745) + (0.0647, 0.0675) ${x}_{1}$+ (− 0.0004, 9.8 × ${10}^{-14}$) ${x}_{2}$+ (0.0001, 6.1 × ${10}^{-14}$) ${x}_{3}$+ (0.0012, 0.0020)${x}_{4}$
FR	*y* = (0.9804, 0.3727) + (− 0.7035, 0.4971) ${x}_{1}$+ (− 0.0013, 0) ${x}_{2}$+ (0.0005, 0.0008) ${x}_{3}$+ (0.0147, 0)${x}_{4}$
FR with nonlinear structure	*y* = (0.7411 0.0497) + (0.0037, 0) ${x}_{3}$+ (− 0.0028,3.0 × ${10}^{-5}$) ${x}_{2}$+ (− 0.8479, 0.0319)${x}_{1}{x}_{4}$ + (0.0557, 0.0069) ${x}_{1}$+ (0.0027, 0)${x}_{4}^{2}$
*Validation3*	
FLSR	*y* = (0.0928, 0.0528) + (0.0714, 0.0458) ${x}_{1}$+ (− 0.0006, 0.0003) ${x}_{2}$+ (0.0001, 0.0005) ${x}_{3}$+ (0.0012, 0.0007)${x}_{4}$
FR	*y* = (0.6232, 1.0521) + (− 0.5403, 0) ${x}_{1}$+ (− 0.0022, 0) ${x}_{2}$+ (0.0019, 0.0002) ${x}_{3}$+ (0.0214, 0)${x}_{4}$
FR with nonlinear structure	*y* = (0.7974, 0) + (− 0.0077, 0) ${x}_{2}$+ (− 0.4771, 0.1511) ${x}_{1}$+ (7.6 × 10^–6^, 9.6 × ${10}^{-7}$)${x}_{1}^{2}{x}_{2}{x}_{3}{x}_{4}$
*Validation4*	
FLSR	*y* = (0.0693, 0.0506) + (0.0499, 0.0494) ${x}_{1}$+ (− 0.0003, 1.3 × ${10}^{-12}$) ${x}_{2}$+ (0.0001, 1 × ${10}^{-11}$) ${x}_{3}$+ (0.0010, 0.0020)${x}_{4}$
FR	*y* = (0.3024, 0.6948) + (− 0.3255, 0.2330) ${x}_{1}$+ (− 0.0023, 2.6 × ${10}^{-6}$) ${x}_{2}$+ (0.0017, 0) ${x}_{3}$+ (0.0273, 0.0070)${x}_{4}$
FR with nonlinear structure	*y* = (0.7631, 0.1099) + (− 0.7848, 0) ${x}_{2}^{3}$+ (0.0026, 0) ${x}_{1}{x}_{4}$+ (− 1.4 × ${10}^{-7}$, − 7.7 × ${10}^{-8}$) ${x}_{1}$ + (0.0298, 0.0089)${x}_{3}$
*Validation5*	
FLSR	*y* = (0.0845, 0.0651) + (0.0611, 0.0610) ${x}_{1}$+ (0.0010, 6.7 × ${10}^{-12}$) ${x}_{2}$+ (0.0001, 0.0004) ${x}_{3}$+ (0.0013, 0.0012)${x}_{4}$
FR	*y* = (0.6407, 0.6230) + (− 0.5857, 0.2458) ${x}_{1}$+ (− 0.0065, 0) ${x}_{2}$+ (0.0027, 0.0005) ${x}_{3}$+ (0.0271, 0.0126)${x}_{4}$
FR with nonlinear structure	*y* = (0.5620, 0.0032) + (− 0.6300, 0.0732) ${x}_{1}$+ (0.0391, 0.0132) ${x}_{2}^{2}{x}_{3}$+ (− 3.4 × 10^–7^,1.8 × ${10}^{-7}$) ${x}_{4}$+ (0.0020, 0)${x}_{1}{x}_{3}$

From the model results presented in the table, it can be concluded that the models developed based on FLSR and FR present the linear fuzzy model form, which only contains the first-order terms. In contrast, the models based on the fuzzy regression with nonlinear structure contain not only first-order terms but also interacted, second-order and even higher-order terms. On the other hand, based on the fuzzy coefficients in the model structure, it can be found that creating a customer preference model based on FLSR, FR, and the proposed method can explain the fuzziness of the modeling.

The suitability of the five methods for modeling consumer preferences can be assessed more intuitively using the values of MRE and MSC, which are shown in Table 3, where a lower value of MRE indicates a higher predictive power of the generated model, and a higher figure of MSC indicates a more reliable and less uncertainty model built by the proposed method. Table 4 shows that the proposed method produces lower average MRE values and higher average MSC values in the experiments compared to the other four methods, indicating a better fit of the proposed method.

**Table 3:** The values of MRE and MSC

Validation tests	Test data	Metrics	FLSR	FR	ANFIS	K-means-ANFIS	FR with nonlinear structure
Validation1	1	MRE	2.4270	3.1215	2.0242	8.0098	0.0034
	2	MSC	1.3103	0.8220	1.2202	0.3019	7.1717
Validation2	3	MRE	1.2312	1.5386	0.5247	6.5903	0.0633
	4	MSC	3.6278	0.8720	1.5572	0.5492	6.9695
Validation3	5	MRE	3.0872	1.0995	0.4756	0.8933	0.2363
	6	MSC	3.3855	0.8728	1.2971	0.5963	5.3052
Validation4	7	MRE	0.4564	0.3102	0.8102	1.1872	0.3962
	8	MSC	0.4375	0.8877	0.4097	0.2807	3.5554
Validation5	9	MRE	4.3572	8.3993	2.0483	2.4139	0.1751
	10	MSC	2.8610	0.6695	1.0589	0.7220	4.0722

**Table 4:** The mean values of MRE and MSC

Mean values	FLSR	FR	ANFIS	K-means-ANFIS	FR with nonlinear structure
Mean MRE	2.3118	2.8938	1.1766	3.8189	0.1749
Mean MSC	2.3244	0.8248	1.1086	0.4900	5.4148

## Discussion

In response to research on consumer preferences regarding online reviews, this paper adopts a fuzzy regression approach with nonlinear structure based on online reviews to build an explicit consumer preference model. The specific solutions are as follows: (1) The polynomial structure of the model built using an MCO algorithm can solve the problem of the high degree of nonlinearity presented in the modeling. (2) The complex fuzzy relationship between consumer preferences and product design attributes can be resolved by identifying the fuzzy coefficients in the generated structure with the application of fuzzy regression. (3) The generated model has an explicit structure, which can be explained by the polynomial structure and the coefficients of each item.

In addition, some limitations are involved in this study which can be divided into three aspects: the experimental preparation before the model construction, the model construction stage, and the completion of the model construction. In the experimental preparation, the main part is the collection and collation of the data set. As the collected comment data may be mixed with duplicate comments, semantically unknown comments, false and invalid comments, etc., the data cleaning task needs to be completed carefully during the data preparation work. As these comments can affect the value of the sentiment score, a certain degree of filtering of invalid information can reduce the inaccuracy of the sentiment score. Therefore, the data preparation process needs to be further strengthened to improve the accuracy of the sentiment score calculation. Then, regarding the stage of model construction, attention needs to be paid to the model parameters settings regarding the number of iterations in MCO, the number of elements in chaotic variables, and the h-value in fuzzy regression. The optimization methods can be introduced to make the appropriate settings to enhance prediction accuracy. Finally, after the model was constructed, an explicit nonlinear fuzzy model was established to display the relationship between consumer preferences and product design attributes. However, the internal correlations among the product design attributes were not investigated and involved in the modeling.

## Conclusion

This paper first briefly compares the existing research on consumer preferences based on online reviews, based on which the black box problems in developing consumer preference models using online reviews are investigated. Combining the nonlinearity, fuzziness, and non-explicitness existing in previous models, an explicit consumer preference model generated by a fuzzy regression method with nonlinear structure based on online reviews is constructed. A web crawler was used to crawl the reviews of smartwatch products on the Amazon shopping platform, and the consumer preferences for 10 products were generated with the help of sentiment score calculation of product reviews and LDA topic classification method. To verify the effectiveness of the research method, the proposed method was applied to the existing consumer preference information of smartwatch products to generate a consumer preference model for the dimension "Customer experience". Finally, through five validation tests and the comparison results of the five methods of FLSR, FR, ANFIS, K-means based ANFIS and the proposed method, it was found that the average relative error of the method proposed in this paper is smaller, and the average systematic confidence is higher, which verifies the effectiveness provided by the proposed method in the practical applications.

Concerning future research, it is hoped that the technical aspects can be taken into account. We plan to improve the adoption of sentiment analysis methods and the accuracy of sentiment score calculation for online reviews. The advanced optimization algorithm can be introduced to determine the optimal settings of parameters of the proposed approach to enhance the accuracy of the prediction. Also, based on the developed customer preference models, the product attributes can be optimized to maximize the sentiment scores of the customer preferences, and the best settings of the product attributes for the new products can be obtained. In addition, the study of considering the changes in consumer preferences at different intervals between user reviews can be performed. For example, when users make their first purchase and when they make a second purchase, their sentiment scores can be adopted to capture the tendency of the changes in customer preferences.

## Data Availability

The data used to support the findings of this study can be obtained from the corresponding author upon request.
